# Chronic Chemogenetic Activation of Astrocytes in the Murine Mesopontine Region Leads to Disturbances in Circadian Activity and Movement

**DOI:** 10.3390/ijms26104793

**Published:** 2025-05-16

**Authors:** Baneen Maamrah, Krisztina Pocsai, Bui Minh Hoang, Ali Abdelhadi, Mustafa Qais Al-Khafaji, Andrea Csemer, Cintia Sokvári, Péter Szentesi, Balázs Pál

**Affiliations:** 1 Department of Physiology, Faculty of Medicine, University of Debrecen, H-4012 Debrecen, Hungary; baneen.maamrah@med.unideb.hu (B.M.); deak-pocsai.krisztina@med.unideb.hu (K.P.); bui.minh.hoang@med.unideb.hu (B.M.H.); aliabdelhadi252@gmail.com (A.A.); mustafaqais@mailbox.unideb.hu (M.Q.A.-K.); csemer.andrea@med.unideb.hu (A.C.); sokvari.cintia@med.unideb.hu (C.S.); szentesi.peter@med.unideb.hu (P.S.); 2 Doctoral School of Molecular Sciences, University of Debrecen, H-4012 Debrecen, Hungary; 3 HUN-REN Cell Physiology Research Group, University of Debrecen, H-4032 Debrecen, Hungary

**Keywords:** astrocyte, pedunculopontine nucleus, mesopontine region, chemogenetics, acoustic startle, activity cycles, gait, spatial memory

## Abstract

We have previously shown that neuromodulatory actions on astrocytes can elicit metabotropic glutamate- and N-methyl-D-aspartate receptor-dependent tonic changes in excitability in the mesopontine region. Although in vitro experiments explored robust effects, the in vivo significance of our findings remained unknown. In this project, chronic chemogenetic activation of mesopontine astrocytes and its actions on movement, circadian activity, acoustic startle and spatial memory were tested. The control group of young adult male mice where mesopontine astrocytes expressed only the mCherry fluorescent tag was compared to the group expressing the hM3D(Gq) chemogenetic actuator. Chronic chemogenetic astrocyte activation reduced the amplitude of the acoustic startle reflex and increased the locomotion speed in the resting period. Gait alterations were also demonstrated but no change in the spatial memory was explored. As a potential background of these findings, chronic astrocytic activation decreased the cholinergic neuronal number to 54% and reduced the non-cholinergic neuronal number to 76% of the control. In conclusion, chronic astrocytic activation and the consequential decrease in the neuronal number led to disturbances in movement and circadian activity resembling brainstem-related symptoms of progressive supranuclear palsy, raising the possibility that astrocytic overactivation is involved in the pathogenesis of this disease.

## 1. Introduction

The mesencephalic locomotor region (MLR) is composed of the pedunculopontine, cuneiform and precuneiform nuclei (PPN, CnF, PrCnF, respectively) [1,2]. The PPN participates in spatial preference, reward-driven movements and sensorimotor gating (filtering of sensory input) in mice [3,4]. In contrast, the PrCnF and the CnF have a more direct role in regulating movement [5,6]. MLR neurons are responsible for regulating different features of movement. Cholinergic neurons of the PPN act on muscle tone and modulate the acoustic startle reflex [7,8,9], glutamatergic neurons of the PPN are associated with explorative movements whereas CnF glutamatergic neuronal activity is proportional to the velocity of movement [6,10,11]. The PPN is also a part of the reticular activating system, which contributes to regulation of the activity cycles and global brain states related to REM sleep and wakefulness by sending cholinergic and non-cholinergic projections to various subcortical locations [12,13]. Excitotoxic damage to the posterior PPN led to learning deficits and alterations in the locomotor activity [14,15]. These changes can be attributed to the lesions of the non-cholinergic neurons as the selective lesion of cholinergic neurons resulted in neither learning impairment nor changes in the locomotor response to nicotine [16]. Instead, sensorimotor and gait deficits developed [17].

The PPN is not only the source of fibers responsible for neuromodulation but also a target of various neuromodulatory actions. Using slice electrophysiology, our laboratory previously demonstrated that various neuromodulatory actions including cholinergic, serotonergic and cannabinoid actions induce astrocytic activation, which uniformly depolarizes or hyperpolarizes neuronal populations determined by their sets of metabotropic glutamate receptors (mGluRs) and regulates phasic, N-methyl-D-aspartate receptor (NMDAR)-mediated depolarization in an activity-dependent manner [18,19,20,21]. Although this action was robust under in vitro conditions, we could not even predict the real impact of the astrocytic activation in the region on the behavioral parameters of mice.

The aim of this project was to have an insight into the pathophysiological significance of regional astrocytic activation. It was previously demonstrated that tau protein expression and a decline in the cholinergic neuronal number in the region lead to a decrease in the startle reflex amplitude and various other gait alterations [17,22]. These conditions well modeled the brainstem-related symptoms of progressive supranuclear palsy (PSP) [22]. In this project, we demonstrated that chronic artificial astrocytic activation led to a reduced acoustic startle reflex and alterations in gait and circadian activity, but the spatial memory was not affected. The background of the finding is the significant reduction in the cholinergic neuronal number. Similarities might be seen between the early symptoms of PSP and our findings, indicating that astrocytes might be involved in the pathogenesis of this neurological disease.

## 2. Results

### 2.1. Two-Bottle Preference Test

As the first preliminary experiment, a two-bottle preference test was performed to measure the average water consumption of the mice and to check whether mice drink the same volume of water if the compound activating DREADD (clozapine-N-oxide hydrochloride; CNO) is dissolved in it. When the two leakage-free drinking bottles were filled with tap water, no significant difference was found in water consumption between the bottles (left bottle: 15.9 ± 8.8 mL/3 days; right bottle: 24.6 ± 10.1 mL/3 days; n.s.). On average, mice consumed 6.75 mL of water daily. As the first four mice weighed 26.96 ± 2.93 g, 4 µg/mL CNO was calculated for the 1 mg/body kg daily CNO consumption. When one bottle contained tap water and 9.6 µM (4 µg/mL) CNO dihydrochloride was dissolved in the other one, no significant difference between water and CNO mixed with water was revealed (tap water: 18.66 ± 3.84 mL/3 days; CNO: 20 ± 6.93 mL/3 days, n.s.). Firstly, based on these data, the concentration of CNO applied in drinking water to reach the 1 mg/kg consumption was determined. As the average body weight of the further 16 mice was 25.7 ± 3.33 g, the calculated daily CNO exposure was slightly higher as planned (1.04 mg/kg). Secondly, no preference or avoidance towards CNO consumption was explored. A similar protocol for chronic administration of CNO in the drinking water was applied by Zhan et al. [23].

### 2.2. Acoustic Startle

**Figure 1:**
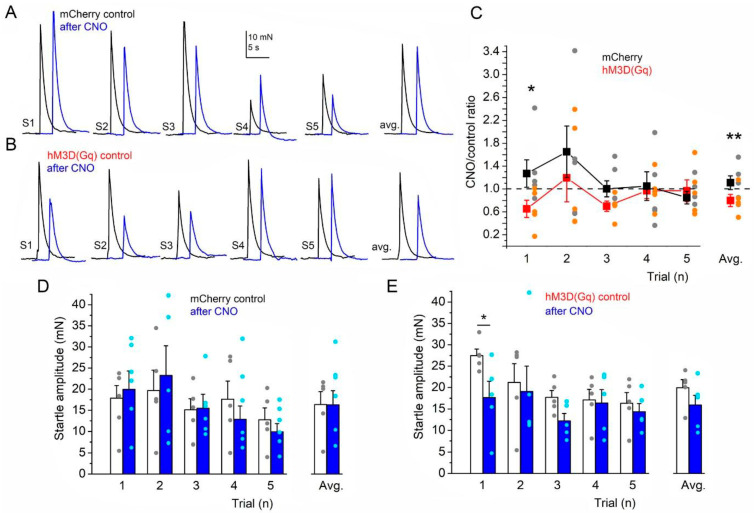
The amplitude of the acoustic startle reflex decreased after chronic astrocyte activation. (**A**). Representative startle traces repeated with 1 min intervals (S1–S5) and their average before (black) and after CNO treatment (blue) of mCherry-expressing control mice. (**B**). Representative startle traces of hM3D(Gq)-expressing mice with the same arrangement as on panel (**A**). (**C**). Statistical comparison of startle amplitudes after CNO normalized to S1 amplitudes before the treatment (CNO/control ratio) during the five concomitant trials and on average. Red traces: average ± SEM of hM3D(Gq)-expressing mice. Orange dots: individual data. Black traces: average ± SEM of mCherry-expressing mice. Gray dots: individual data. (**D**). Statistical comparison of absolute startle amplitudes before (hollow columns: average ± SEM, gray dots: individual data) and after CNO treatment (blue columns: average ± SEM, light blue dots: individual data) along the five trials and on average in mCherry-expressing controls. (**E**). Statistical comparison of absolute startle amplitudes before and after CNO treatment in hM3D(Gq)-expressing mice with the same arrangement as on panel D (n = 8 for the DREADD group and 7 for the control group); * *p* < 0.05; ** *p* < 0.01.

In this experiment, the amplitude of the acoustic startle reflex and its short-term habituation were tested (Figure 1A,B). When the responses after CNO drinking were normalized to the amplitudes before the treatment, a significantly lower first relative amplitude was detected in the DREADD group compared to the control group (first amplitude: 127 ± 24% with CNO treatment in the control group and 65.3 ± 14.9% with CNO treatment in the DREADD group; *p* = 0.012). Similarly, the average normalized amplitude of the five concomitantly measured startle responses showed a significant decrease in the DREADD group compared to the control group (average amplitude: 111 ± 12% with CNO treatment in the control group and 79.9 ± 10.6% with CNO treatment in the DREADD group; *p* = 0.006; Figure 1C). In the control (mCherry) group, the first startle amplitude was 17.88 ± 2.98 mN under the control conditions, which became 19.96 ± 2.98 mN after CNO drinking (n.s.). However, the further amplitudes did not show any changes (DREADD group: 2nd stimulus: 21.2 ± 4.39 mN before and 19.09 ± 5.92 mN after CNO consumption; 3rd: 17.69 ± 1.63 mN before and 12.19 ± 1.75 mN after CNO; 4th: 17.1 ± 2.49 mN before and 16.38 ± 3.13 mN after CNO; 5th: 16.33 ± 2.5 mN before and 14.36 ± 1.99 mN after CNO; mCherry group: 2nd stimulus: 19.69 ± 4.83 mN before and 23.24 ± 7.04 mN after CNO consumption; 3rd: 15.12 ± 2.6 mN before and 15.49 ± 3.32 mN after CNO; 4th: 17.62 ± 4.31 mN before and 12.89 ± 3.13 mN after CNO; 5th: 12.75 ± 2.81 mN before and 9.91 ± 1.94 mN after CNO). In the DREADD group, the average first amplitude was 27.48 ± 1.52 mN, which decreased to 17.64 ± 3.82 mN after the CNO treatment (*p* = 0.021; Figure 1D,E). In conclusion, the first acoustic startle reflex itself was declined after chronic astrocytic stimulation, but the short-term habituation did not have any significant change.

### 2.3. Circadian Activity

**Figure 2:**
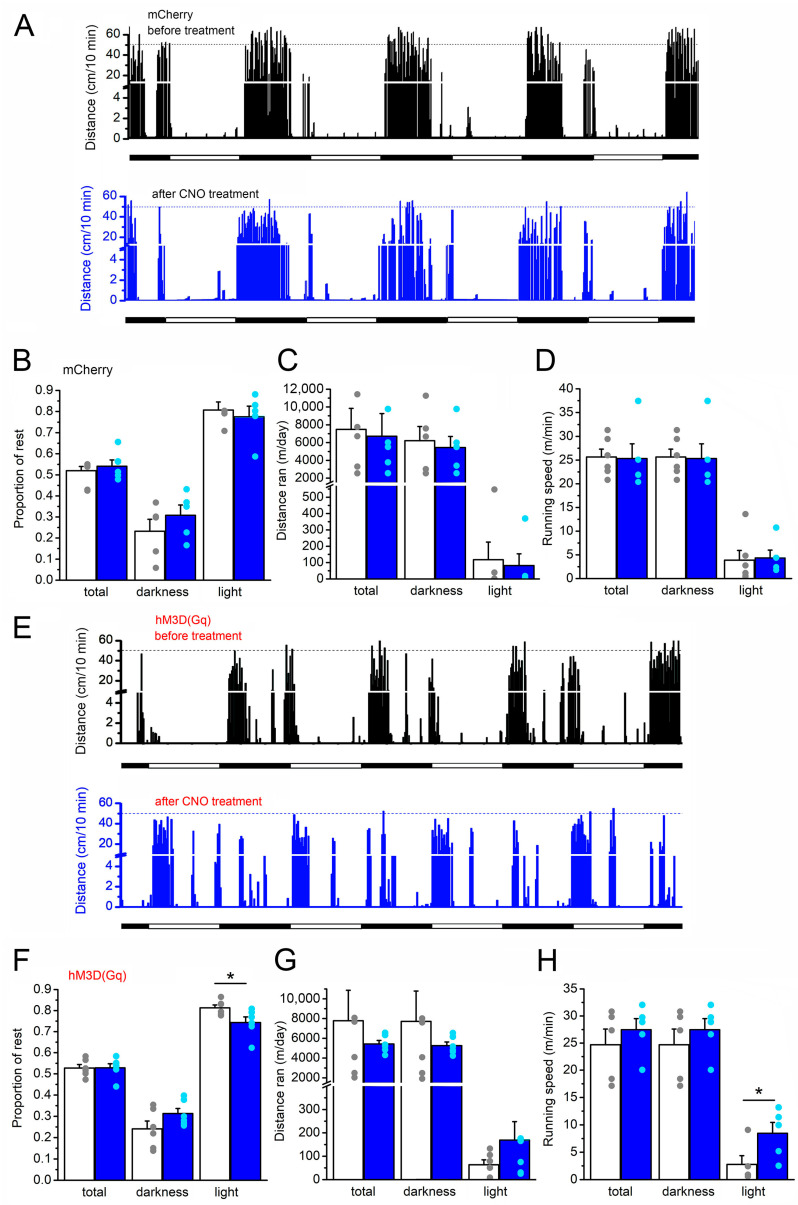
The circadian activity of mice was altered by the mesopontine astrocytic overactivation. (**A**). Activity pattern of an mCherry-expressing control mouse before (black columns) and after CNO consumption (blue columns). Each bar represents the distance moved in cm in 10 min bins. Horizontal bars below the graph represent light (hollow) and dark (black) environmental conditions. (**B**). Statistical comparison of the proportions of time spent at rest before (hollow columns: average ± SEM, gray dots: individual data) and after CNO consumption (blue columns: average ± SEM, light blue dots: individual data) in the whole time (“total”), in the active period (“darkness”) and in the resting period (“light”) in the case of the mCherry-expressing control mice. (**C**). Statistics of the distances moved in a day of mCherry-expressing control mice with similar arrangement as on panel (**B**). (**D**). Statistics of the maximal recorded running speed in a day of mCherry-expressing control mice with similar arrangement as on panel (**B**). (**E**). Activity pattern of an hM3D(Gq)-expressing mouse in a similar arrangement as on panel (**A**). (**F**). Statistical comparison of the proportions of time spent at rest before and after CNO consumption of the hM3D(Gq)-expressing mice. (**G**). Statistics of the distances moved in a day of the hM3D(Gq)-expressing mice. (**H**). Statistics of the maximal recorded running speed in a day of the hM3D(Gq)-expressing mice. Panel arrangements are similar to those on panels (**B**) and (**D**) (n = 6 for the DREADD group and 6 for the control group); * *p* < 0.05.

Next, the circadian activity of mice was tested by analyzing the relative time spent at rest, the distances run, as well as the maximal running speed in the resting light period and in the active dark period. In the control group, there was no change in the percentage of time spent in inactivity in darkness (active period, 23 ± 5.7% before and 30.8 ± 4.9% after CNO drinking), light (resting period, 80.7 ± 3.8% before and 77.5 ± 5% after CNO drinking) and in total (52 ± 2.3% vs. 54 ± 3%, respectively; Figure 2A,B). Similarly, no difference was seen in distances traveled per day (darkness: 6215 ± 1602 m/day before and 5540 ± 1255 m/day after CNO drinking; light: 117 ± 107 m/day before and 81.5 ± 72 m/day after CNO drinking; total: 6347 ± 1607 m/day before and 5522 ± 1228 m/day after CNO drinking) of the mCherry-expressing mice (Figure 2C). The maximal running speed of the mice was also not affected by the CNO treatment in the control group (darkness: 25.6 ± 1.66 m/min before and 25.3 ± 3.1 m/min after CNO drinking; light: 3.9 ± 2 m/min before and 4.3 ± 1.6 m/min after CNO drinking; Figure 2D).

In contrast, certain activity parameters were altered in the DREADD group expressing hM3D(Gq) (Figure 2E). The percentage of inactivity during the active period (darkness) was not altered significantly (24.1 ± 3% before and 31.3 ± 2%; Figure 2F). In contrast, when illuminated (in the inactive period), the time spent at rest was significantly declined (81.3 ± 1.4% before and 74.3 ± 2% after CNO consumption; *p* = 0.02; Figure 2F). The total activity was not significantly altered (52.7 ± 1.6% vs. 52.8 ± 1.9%, Figure 2F). As seen in the control group, no change was seen in distances run daily in the DREADD group (darkness: 7713 ± 3076 m/day before and 5259 ± 369 m/day after CNO drinking; light: 63.6 ± 21 m/day before and 169.1 ± 79.2 m/day after CNO drinking; total: 7776.6 ± 3083 m/day before and 5428 ± 354 m/day after CNO drinking; Figure 2G). The maximal running speed of the mice was significantly increased by the CNO treatment in the DREADD group during the illuminated (resting) period (darkness: 24.7 ± 2.89 m/min before and 27.47 ± 2.05 m/min after CNO drinking; light: 2.77 ± 1.61 m/min before and 8.45 ± 1.99 m/min after CNO drinking; Figure 2H). In conclusion, mice in the DREADD group became more restless in the resting period (when the cages were illuminated), whereas the control group was not affected.

### 2.4. Gait Alterations

**Figure 3:**
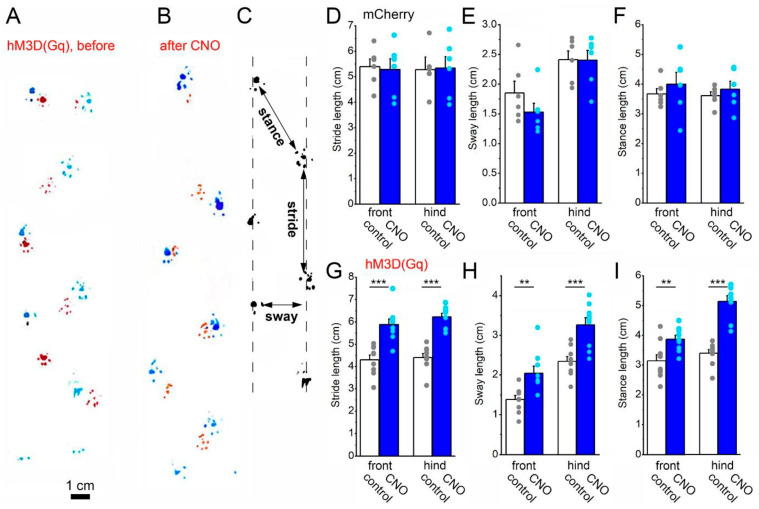
Alterations in the movement pattern after astrocytic overactivation. (**A**,**B**). Representative footprint patterns of a hM3D(Gq) mouse before (**A**) and after CNO treatment (**B**). Blue: hind paws, red: front paws. (**C**). Measurement of footprint parameters. (**D**–**F**). Statistical summary of stride, sway and stance lengths before (hollow columns: average ± SEM, gray dots: individual data) and after CNO treatment (blue columns: average ± SEM, light blue dots: individual data) of mCherry-expressing control mice. (**G**–**I**). Statistical summary of stride, sway and stance lengths of hM3D(Gq)-expressing mice with a similar arrangement as on (**D**–**F**) (n = 9 for the DREADD group and 6 for the control group); ** *p* < 0.01; *** *p* < 0.001.

In this experiment, gait alterations were aimed to be assessed by using the footprint test. Parameters of the footprint, such as the stride length, sway length and stance length, were investigated and compared before and after the CNO treatment in both animal groups (Figure 3A–C). No difference was seen in these parameters in the mCherry-expressing control group (front limb: stride length: 5.39 ± 0.3 cm before and 5.27 ± 0.4 cm after CNO; sway length: 1.84 ± 0.19 cm before and 1.53 ± 0.15 cm after CNO; stance length: 3.67 ± 0.18 cm before and 3.99 ± 0.4 cm after CNO; hind limb: stride length: 5.27 ± 0.35 cm before and 5.33 ± 0.46 cm after CNO; sway length: 2.41 ± 0.15 cm before and 2.39 ± 0.17 cm after CNO; stance length: 3.61 ± 0.13 cm before and 3.83 ± 0.27 cm after CNO (Figure 3D–F). In contrast, the DREADD group expressing hM3D(Gq) in mesopontine astrocytes displayed a significant increase in all detected parameters (front limb: stride length: 4.3 ± 0.22 cm before and 5.88 ± 0.25 cm after CNO, *p* = 0.000116; sway length: 1.37 ± 0.1 cm before and 2.03 ± 0.18 cm after CNO, *p* = 0.0028; stance length: 3.13 ± 0.21 cm before and 3.86 ± 0.13 cm after CNO, *p* = 0.0053; hind limb: stride length: 4.4 ± 0.18 cm before and 6.22 ± 0.16 cm after CNO, *p* < 0.00001; sway length: 2.33 ± 0.12 cm before and 3.26 ± 0.18 cm after CNO, *p* = 0.00039; stance length: 3.39 ± 0.12 cm before and 5.13 ± 0.19 cm after CNO, *p* < 0.00001; Figure 3A,B,G–I). In summary, all investigated parameters (as stride, sway and stance length) increased by chronic astrocytic activation, whereas no significant change was seen in the similarly treated control group.

### 2.5. Spatial Memory

**Figure 4:**
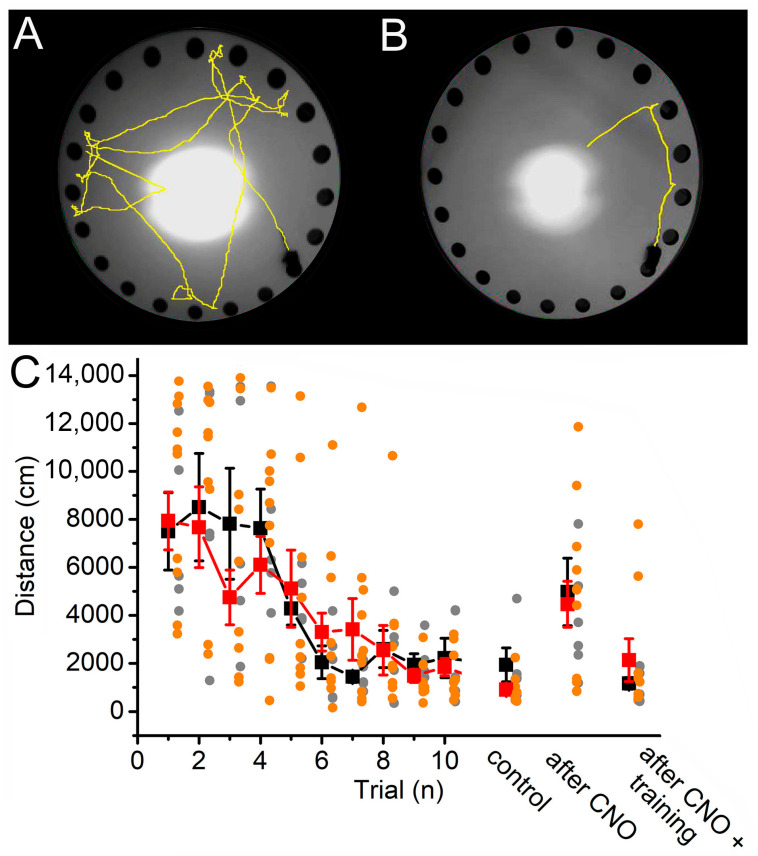
The spatial memory was not affected by mesopontine astrocytic overactivation. (**A**,**B**). The path of the mouse to the shelter in the Barnes maze test (yellow) before (**A**) and after training (**B**). (**C**). Statistical comparison of the distances run during training trials, after 3 days (“control”), directly after CNO consumption and after a second training series. Red squares: average ± SEM, orange dots: individual data of hM3D(Gq) mice. Black squares: average ± SEM, gray dots: individual data of mCherry-expressing control mice (n = 7 for the DREADD group and 7 for the control group).

The Barnes maze task was conducted to investigate the effect of astrocyte overstimulation on spatial memory and learning. Under the control treatment conditions (before CNO drinking), the control and DREADD groups had the same ability to form spatial memory (Figure 4A–C). Two days after the 10th learning session, as well as after CNO drinking and after a new repetition series with 10 sessions, no differences were seen between the two populations (1938.6 ± 706.2 cm in the control group and 906 ± 189.3 cm in the DREADD group at the end of the training session before CNO drinking; 4978.7 ± 1409 cm in the control group and 4463.6 ± 959 cm in the DREADD group directly after CNO drinking; 1166.1 ± 240.3 cm in the control group and 2136 ± 894 cm in the DREADD group after the second series of training; Figure 4C). In conclusion, no significant change was observed between the mCherry-expressing control and hM3D(Gq)-expressing DREADD group.

### 2.6. Evaluation of the Injection Sites

**Figure 5:**
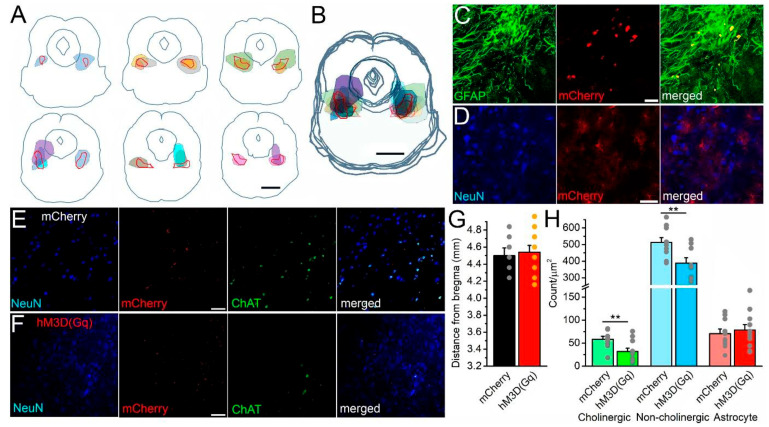
Evaluation of the injection sites and chronic changes in neuronal number. (**A**). Individual coronal sections from 4.26 to 4.96 mm caudal from the bregma by 160 µm. Gray lines: contours of the brainstem, the aqueduct and the periaqueductal gray. Red contours: pedunculopontine nucleus. Colored areas: individual places of bilateral mCherry expression from all experiments. Scale bar: 1 mm. (**B**). Merged image of six coronal mesencephalic sections on panel (**A**). Scale bar: 1 mm. (**C**). Colocalization of GFAP immunohistochemical labeling and mCherry expression on a single 1 µm thick z-stack image (left, green: GFAP immunopositivity, middle, red: mCherry expression, right: merged image). Scale bar: 20 µm. (**D**). The lack of colocalization in NeuN labeling and mCherry expression on a single z-stack image (left, blue: NeuN labeling, middle, red: mCherry expression, right: merged image). Scale bar: 20 µm. (**E**). Cholinergic and non-cholinergic neuronal numbers after chronic CNO treatment and behavioral tests in mCherry-expressing control mice on a single z-stack image (from left to right: blue: NeuN labeling, red: mCherry expression, green: ChAT immunohistochemistry, merged). Scale bar: 50 µm. (**F**). Cholinergic and non-cholinergic neuronal numbers after the same chronic CNO treatment and behavioral tests in hM3D(Gq)- and mCherry-expressing DREADD mice on a single z-stack image (from left to right: blue: NeuN labeling, red: mCherry expression, green: ChAT immunohistochemistry, merged). Scale bar: 50 µm. (**G**). Statistical comparison of the injection sites caudal from the bregma in mm in the case of control mice (mCherry, black columns: average ± SEM; gray dots: individual data) and DREADD mice (hM3D(Gq), red columns: average ± SEM; orange dots: individual data). (**H**). Statistical comparison of cholinergic neuronal numbers after CNO treatment in control mice (mCherry, light green columns) and in DREADD mice (hM3D(Gq), green columns); non-cholinergic neuronal numbers (control: light blue, DREADD: blue) and astrocytes (control: pink, DREADD: red). Columns represent average ± SEM, gray dots indicate the individual data points (n = 8 for the DREADD group and 6 for the control group); ** *p* < 0.01.

The center of the injection site targeted the pedunculopontine nucleus (PPN) 4.26–4.96 mm caudal from the bregma. However, the mCherry expression in astrocytes was not limited to the PPN but neighboring areas were also involved. Expression was seen in the dorsal one-third of the oral pontine reticular nucleus, in the middle region of the mesencephalic reticular formation, the retrorubral field and in the retrorubral nucleus, the precuneiform nucleus, the lateral and ventrolateral periaqueductal gray, the lateral part of the dorsal raphe nucleus, the medial paralemniscal nucleus, the microcellular tegmental nucleus and the ventral part of the cuneiform nucleus (Figure 5A,B) [24]. The mCherry tag was expressed in both the control and DREADD groups. All mCherry-positive somata partially or fully overlapped with the GFAP immunohistochemistry (Figure 5C), whereas NeuN-positive somata did not overlap with mCherry-positive somata (Figure 5D). Of note, several cell bodies and processes with GFAP positivity but no mCherry labeling were seen and mCherry-positive processes often surrounded NeuN-positive somata.

### 2.7. Histological Analysis

In parallel with the evaluation of the injection site and the involved brain areas, the cholinergic, non-cholinergic and astrocytic numbers were counted on the basis of ChAT immunopositivity, NeuN labeling and mCherry expression. Somata with ChAT positivity and NeuN labeling were considered as cholinergic neurons, ChAT-negative and NeuN-positive ones were counted as non-cholinergic neurons, whereas mCherry-positive somata were considered as astrocytes. The evaluation was performed on both sides of the coronal plane at the injection site. Cells of a 0.43 × 0.43 mm square with a center on the PPN were counted (Figure 5E,F). The coronal plane where the cell counting took place was 4.5 ± 0.09 mm caudal from the bregma in the control group exclusively expressing the mCherry tag in the astrocytes and 4.54 ± 0.08 mm in the DREADD group expressing the mCherry and chemogenetic actuator together, showing no significant difference between the two groups (Figure 5G).

After chronic CNO treatment, the control group had 58.62 ± 6.2 cholinergic, 512.89 ± 28.72 non-cholinergic and 70.61 ± 10.02 astrocytic somata per mm^2^. In the DREADD group, 31.89 ± 7.03 cholinergic, 389.06 ± 31.9 non-cholinergic and 78.19 ± 12.17 astrocytic somata per mm^2^. In summary, chronic chemogenetic astrocytic activation reduced the cholinergic neuronal number to 54.4% of the control (*p* = 0.0054), the non-cholinergic neuronal number was 75.8% of the control (*p* = 0.0049), whereas the astrocytic number of the DREADD group was 110.7% of the control (*p* = 0.32; Figure 5H).

## 3. Discussion

Chronic chemogenetic activation of astrocytes in the PPN and neighboring mesopontine areas led to a decrease in the acoustic startle reflex, decreased the time spent at rest and increased the maximal speed of movement. The stride, sway and stance lengths of both the front and hind limbs increased. The spatial memory was not affected. *Post hoc* histological experiments revealed that the number of cholinergic neurons significantly decreased in the group with chronic artificial astrocyte activation in contrast to the control group similarly operated on and treated with CNO but lacking astrocytic overactivation.

We previously identified an astrocyte-dependent common component of several (cholinergic, cannabinoid, serotonergic and partially orexinergic) neuromodulatory mechanisms using in vitro slice electrophysiology and imaging experiments [18,19,20,21]. We found that a subpopulation of neurons were hyperpolarized and had a decreased firing rate via group I. mGluR activation, whereas another subgroup was depolarized with an increased firing rate via group II. mGluRs after astrocytic activation by neuromodulatory actions or optogenetics [18,20]. A third subgroup did not respond to astrocytic stimulation. Another regulatory mechanism related to neuromodulatory agents was via the extrasynaptic, GluN2B subunit-containing NMDARs. If ‘slow inward currents’ (SICs) had a low frequency under control conditions, neuromodulatory actions uniformly increased this frequency, whereas if the SIC frequency was initially high, the same neuromodulatory agonists decreased it. This action was due to NMDAR activation and inactivation [21]. Although the actions were detected on in vitro preparations, one could not even predict the (patho)physiological significance of these findings of the explored mechanisms. To address this problem, we conducted in vivo, behavioral studies to evaluate the potential impact on artificial astrocytic overactivation on functions related to the MLR and related brain areas.

Chemogenetic activation of astrocytes of various brain locations in behavioral tests has been performed with several experimental arrangements. Acute Gq-mediated DREADD activation of the hippocampal and medial prefrontal cortical astrocytes improved memory and learning [25,26,27]. Similar acute astrocytic DREADD activation affected reward and addictive behavior in the nucleus accumbens [28], led to a shift from habitual action to goal-directed behavior in the striatum [29] and reduced fear expression in the amygdala [30]. Acute chemogenetic activation of pontine astrocytes suppressed REM sleep and decreased the number of REM periods [31].

Chronic chemogenetic activation (varying between 7 days and lifelong application) of the hippocampal astrocytes affected fear memory in a different way than neuronal activation [32] or affected excitotoxicity in a contradictory way. It led to morphological changes in astrocytes and neuroinflammatory phenotype accompanied by cognitive decline [33]; or, in contrast, it reversed the kainate-induced increase in metabolism and prevented excitotoxic damage and decline in memory [34].

PSP is a neurodegenerative disease with motor symptoms resembling Parkinson’s disease. Symptoms affecting gait and posture are combined with behavioral and cognitive symptoms including dementia and motor abnormalities associated with REM sleep, as well as the lack of the acoustic startle reflex [35,36]. The disease is also characterized by neuronal, astroglial and oligodendroglial tau protein accumulation, which is localized to the brainstem and the subcortical structures in the early phase of the disease in the case of the neurons and involves cortical areas with the progress [37].

Previous studies demonstrated that excitotoxic damage to the posterior PPN caused learning deficits and alterations in locomotor responses [14,15]. It was later shown that these alterations were consequences of actions on non-cholinergic neurons, as the depletion in the cholinergic neurons did not lead to similar symptoms [16]. The brainstem-related symptoms of PSP were modeled in rodents by manipulations on the cholinergic brainstem [17,22]. Firstly, selective depletion in the cholinergic neurons of the PPN mimicked the motor symptoms of PSP such as the decline in the acoustic startle reflex and motor deficits including the loss of precision in gait regulation [17]. Secondly, expression of tau protein in the PPN led to taupathy with a decline in the cholinergic neuronal number in the PPN and dopaminergic cell loss in the substantia nigra, symptoms overlapping with the ones found in the previous study [22]. As neuroinflammation induced by taupathy and microglial activation can be accompanied by pathological astrocytic activation [38,39,40,41], we filled this gap by showing that chronic astroglial stimulation is able to cause symptoms resembling PSP and PPN excitotoxicity.

Chronic astrocytic activation almost halved the cholinergic cell number and decreased the non-cholinergic neuronal number in a significant but smaller proportion, whereas no difference was seen in the astrocytic cell number. The possible background of this phenomenon is that chemogenetic activation of astrocytes causes glutamate release from astrocytes, which in turn elicits tonic depolarization on PPN neurons possessing group II. metabotropic glutamate receptors [18,20] and phasic depolarization via activation of extrasynaptic NMDA receptors [21]. As a subset of neurons had group I. mGluR-related neuronal hyperpolarization and no tonic current is elicited in a further population, not all neurons of the region are equally vulnerable to astrocytic overactivation [18,20].

A characteristic symptom of chronic astrocytic activation of the MLR and neighboring regions was the decrease in the acoustic startle reflex. This reflex is known to be modulated by the PPN. Optogenetic stimulation of cholinergic neurons enhanced it, whereas non-cholinergic neurons likely modulate it via prepulse inhibition [42]. In line with this observation, selective loss of PPN cholinergic neurons or tau protein expression by them decreased the amplitude of the acoustic startle reflex [17,22]. The clinical significance of the decrease (or disappearance of) in the acoustic startle reflex following midbrain manipulations could be that the acoustic startle reflex fully disappears in PSP, where one of the first structures affected by tau deposition is the brainstem [35,37]. In comparison to the studies where all PPN cholinergic neurons were affected, the decline in the startle reflex in our study is weaker, which seems to correlate with the magnitude of the cholinergic loss.

Hearing loss might also alter the acoustic startle reflex. In cases of partial damage to the cochlear outer hair cells and consequential hyperactivity of the inferior colliculus, the startle amplitude was increased. The startle reflex almost fully disappeared in cases if the outer hair cells were fully destroyed [43,44]. We also demonstrated that partial damage to the cochlea and hyperexcitability of the auditory brainstem lead to an exaggerated startle reflex [45]. As we have not tested it, we cannot fully exclude that hearing loss does not occur in our experimental model. However, it is unlikely that hearing loss has an impact on the changes in the acoustic startle reflex amplitude. Firstly, hyperexcitability of the auditory brainstem—which is the consequence of astrocytic overactivation—rather induces an increase and not decrease in the startle amplitude [18,20,45]. Secondly, the *post hoc* morphological analysis revealed no mCherry (and DREADD) expression of the auditory-related areas.

Alterations in the circadian activity were found as consequences of the chronic activation of midbrain astrocytes. The activity of mice increased in the resting period, as well as the maximal movement velocity also becoming faster. The changes we observed might be consequences of sleep regulation abnormalities. Acute chemogenetic activation of pontine astrocytes suppressed REM sleep and decreased the number of REM periods [31] and the main target of our manipulations was the PPN, which is a known regulator of REM sleep and wakefulness [13]. Another possible explanation is that excitotoxic lesions related to astrocytic overactivation increased locomotion [15]. Astrocytic activity is known to influence locomotion under pathological conditions as acute chemogenetic astrocytic activation via G_i_-coupled DREADD compensated for the locomotor deficits in a Parkinsonian mouse model [46]. Of note, chronic chemogenetic activation of dorsal raphe serotonergic neurons elicited similar changes in circadian locomotion as we observed: an increase in motor activity was detected during the illuminated resting period, whereas the activity was decreased in the dark, active period [47]. The similarities with our work might be due to the similar role of the dorsal raphe in the regulation of activity cycles or it might be the consequence of indirect activation of raphe neurons (via the alterations in PPN activity) or direct actions on it (DREADD expression partially involving the dorsal raphe).

When analyzing the footprint pattern, we found that all measured footprint parameters (stride, stance and sway length of the front and hind paws) were increased. A depletion in PPN cholinergic neurons elicited marked motor deficits, such as slips of the rear paw and loss of precision in gait regulation [17,22]. Several diseases and disease models affect the stride and sway length. The stride length is shorter and the width is narrower in hyperekplexia accompanied by increased muscle tone [48], as well as in an animal model of Alzheimer’s disease [49]. In the latter case, the walking speed was reduced as well. In PSP, the stride length is also shorter but the step width is significantly wider [50]. Our findings showing an overall increase in footprint measures do not fit any of the disease symptoms above. A possible explanation might be that the movement velocity directly correlates with the stride length [51] and we found an increase in the movement speed with the activity wheel test. Therefore, we might observe a combination of PSP-like symptoms (increased sway length) and consequences of faster walking (increased stride length).

Excitotoxic lesions of the PPN (predominantly due to actions on non-cholinergic neurons) elicited learning [14,16] and attention deficits [52]. Memory and attention deficits are also seen in PSP [35] and inappropriate phasing of sleep and circadian disturbances might also cause memory disturbances [53]. Based on these data, we set the Barnes maze test as the one fitting best in the sequence of tests least affecting the stress level of mice (in contrast to fear conditioning or swim tests). We found no difference in the control and DREADD groups, which is consistent with the previous findings that the decline in cholinergic neurons does not critically affect memory and the number of non-cholinergic neurons (which potentially affect memory) was less severe during our testing period. In our experimental arrangement, almost half of the cholinergic neurons were lost (54% of the control remained), whereas the non-cholinergic neurons suffered from a reduction to three-fourths of the control (76% of the control existed). Testing memory functions with a more prolonged CNO consumption or a longer time after the treatment or employing alternative tests of memory and attention would potentially reveal further symptoms.

Although our project demonstrates unambiguous changes in several motor functions after overactivation of astrocytes, there are some caveats to take into account when conclusions are drawn. Firstly, although the aim was to overactivate astrocytes in the MLR with a center in the PPN, the labeling was not restricted exclusively to the region but the related neighboring areas also showed mCherry expression. Arguing for the significance of our findings, we might point out that we found a stronger decline in the number of ChAT-positive neurons. It might indicate that the main change in the background of the behavioral alterations was the effect on the cholinergic neurons; however, alterations in the non-cholinergic neuronal number of the MLR and regions distinct from it should also be considered. Secondly, CNO consumption of mice was voluntary and based on the preliminary data of water consumption. This way of administration was the least traumatizing for the experimental animal in contrast to the possibility of the daily intraperitoneal injection or depo injection of CNO, which would alter the behavior due to the avoidance of stress by touching, or by abdominal pain. We assume that administering exactly the same amount of CNO to mice would lead to clearer results with a smaller standard error; thus, we potentially underestimated the actions we described. Thirdly, it is known that CNO itself affects some behavioral parameters in *naïve* mice. The amplitude of the acoustic startle was shown to be reduced and the NREM-REM sleep latency and episode duration of NREM sleep before REM onset were altered by the same concentration of CNO as we used (1 mg/kg) [16,54]. Higher doses of CNO attenuated amphetamine-induced hyperlocomotion and changed the NREM and REM sleep duration but did not affect spontaneous locomotion (5–10 g/kg) [16,54,55]. Addressing this concern, we designed the experiments to exclude these actions of CNO or its conversion to clozapine [55]. To achieve this, we compared all findings on DREADD mice to the controls undergoing the same operation and CNO treatment without DREADD expression. In addition, no reduction in the startle amplitude was seen in the control and significant alterations in gait or circadian activity were also absent.

In summary, chronic astrocytic activation in the PPN and the neighboring mesopontine regions led to a strong reduction in cholinergic and moderate but still significant decrease in non-cholinergic neuronal populations. This alteration in the neuronal number was likely the consequence of the exaggerated astrocytic glutamate release and the consequential overstimulation of neuronal mGluRs and NMDARs. This neuronal loss induced a decrease in the acoustic startle reflex, alterations in the circadian rhythm and restlessness, which partially model the brainstem symptoms of PSP. Our results also suggest that prevention of astrocytic overactivation or neuronal overstimulation by glutamate might serve as a potential therapeutic target for stopping or slowing the progress of neurodegenerative diseases.

## 4. Materials and Methods

### 4.1. Experimental Animals

The experiment was conducted on 16 male C57BL/6 wild-type and 4 male ChAT-tdTomato young adult (2-month-old) choline acetyltransferase (ChAT)-tdTomato mice with a B6 background-expressing tdTomato fluorescent marker under ChAT promoter to label cholinergic neurons [55]. ChAT-tdTomato mice were bred in our own animal facility by crossing homozygous ChAT-cre (B6;129S6-Chat^tm2(cre)Lowl/^J; Jax number: 006410) and floxed-stop- tdTomato (B6;129S6-Gt(ROSA)26Sor^tm9(CAG-tdTomato)Hze/^J; Jax mice accession number: 007905) strains purchased from Jackson Laboratories (Bar Harbor, ME, USA). Mice were kept under normal dark–light cycles and standard animal facility conditions and housed together with their littermates. Food and water were available ad libitum.

The experiments were performed following the appropriate national and international (EU Directive 2010/63/EU for animal experiments) and institutional guidelines and laws on the care of research animals. The experimental protocols were approved by the Hungarian National Food Chain Safety Office (HB/06-ÉLB/129-1/2020; 19/2019/DEMÁB, HB/15-ÉLB/00136-42/2023; 3/2023/DEMÁB; 17/2023/DEMÁB).

In total, 20 mice were operated on; 13 of them were designated as a DREADD group expressing hM3D(Gq) and mCherry tag in the mesopontine astrocytes, and another 7 as a control group exclusively expressing the mCherry tag. Nine of the DREADD group and all the control mice were wild-type C57BL/6 mice, whereas four from the DREADD group were ChAT-tdTomato mice. We involved 16 mice in all behavioral tests except the two-bottle preference test; the rest of the DREADD mice, which were ChAT-tdTomato (n = 4), underwent the preliminary drug preference test. However, some measurements had to be excluded because of technical reasons. In Table 1, the number of mice used for various behavioral experiments is represented.

### 4.2. Surgery

The aim of the surgery was the microinjection of virus vectors carrying plasmids to the mesopontine region with a center in the pedunculopontine nucleus (PPN). In the DREADD group, an AAV virus vector carrying a plasmid-encoding hM3D(Gq) chemogenetic actuator and mCherry fluorescent tag expressed under a GFAP promoter was injected (pAAV-GFAP-hM3D(Gq)-mCherry (AAV5); a gift from Bryan Roth; Addgene plasmid # 50478; http://n2t.net/addgene:50478 accessed on 14 May 2025; RRID:Addgene_50478). In the control group, a plasmid exclusively containing the mCherry fluorescent tag was injected (pAAV-GFAP104-mCherry (AAV5), a gift from Edward Boyden; Addgene plasmid # 58909; http://n2t.net/addgene:58909 accessed on 14 May 2025; RRID:Addgene_58909).

Mice were anesthetized with the administration of ketamine (100 mg/kg) and xylasine (10 mg/kg) by peritoneal injection. When the anesthesia was complete (monitored by the lack of flexor reflex and blinking reflex), mice were placed on a heating pad maintaining 37 °C body temperature, the head was fixed in a stereotaxic frame (RWD Life Science Co., Ltd., Shenzhen, China) and eyes were covered by eye drops containing hyaluronic acid (Vizol S 0.21% eye drop, Penta Pharma Co., Budapest, Hungary). After opening the skin and performing trepanation with a microdrill (RWD Life Science Co., Ltd., Shenzhen, China), 200 nl of the virus (titer: 2 × 10^13^ GC/mL for pAAV-GFAP-hM3D(Gq)-mCherry (AAV5) and 1.7 × 10^13^ GC/mL for pAAV-GFAP104-mCherry (AAV5)) was injected with a Hamilton syringe and microinjector (RWD Life Science Co., Ltd., Shenzhen, China). The injection was performed bilaterally with the following stereotaxic coordinates: 4.96 mm caudally from the bregma, 1.25 mm from the midline, 3.4 mm depth. After the operation, the skull was closed with bone wax and the skin was sutured (5-0 Vicryl, Ethicon). Mice received ibuprofen for pain relief (30 mg/kg; Nurofen Baby, Reckitt Benckiser Ltd., Budapest, Hungary) after the anesthesia wore off and in the next 2 postoperative days and were kept in individually ventilated cages for 7 days after surgery.

### 4.3. Behavioral Tests

**Figure 6:**
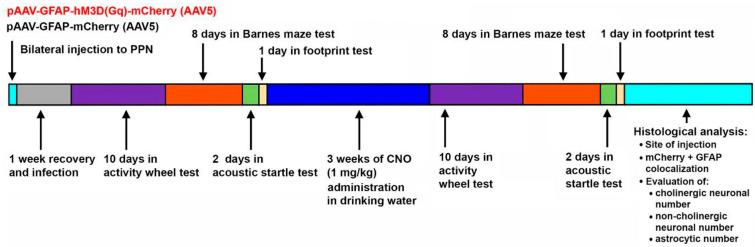
The experimental design and the timing of the operation, treatment and behavioral tests.

All behavioral tests detailed below were performed in a sequence shown in Figure 6: before and after 3 weeks of CNO consumption (Tocris Cookson Ltd., Bristol, UK; 1.04 mg/kg consumed; 4 µg/mL, 9.6 µM in drinking water). The dosage was determined on the basis of the average water consumption measured in the drinking experiments with the two-bottle preference test (see below).

### 4.4. Two-Bottle Preference Test

Mice were placed in cages equipped with 2 drinking bottles. For the initial 3 days of the test, both bottles contained the same tap water and consumption in ml/3 days was measured. For the next 3 days, the red bottle was filled with CNO-containing water (4 µg/mL; 9.6 µM) [23].

### 4.5. Activity Wheel Test

For measuring activity cycles, the distance and maximal velocity of voluntary movements, mice were individually placed in cages equipped with running wheels. Mice spent 10 days in the activity wheel setup from which the first 3 days—as a period for adaptation—were not considered in the evaluation of the results. Mice were exposed to a 24 h light–dark cycle (light from 6AM to 6PM) for a week after one week of habituation under the same conditions. The number of rotations of the activity wheel in 10 min intervals was measured, the distance traveled in m was also detected as well as the maximal running velocity in m/min [56].

### 4.6. Barnes Maze Test

Mice were placed on an elevated circular maze that was surrounded by 20 holes along the edge. The setup was illuminated from above, and 60 dB white noise was applied. An escape box was positioned beneath one hole for the duration of the experiment. Movements of the mouse were recorded with a web camera. Mice were placed at the center of the maze and recording started until they found the shelter. A box was placed over the mice and removed to allow exploration at the beginning of measurements. The experiment included two phases: an acquisition phase and a probing trial. In the acquisition phase, the mice were placed at the center of the maze and allowed to move around until they found the escape box for 5 days, with two sessions per day. After a 3-day retention interval, a probe trial was performed to assess and find the location of the shelter, where it was placed before, with one single trial in this phase [57]. The maze was cleaned between trials to prevent smell interference. We measured the distance run by the mice to find the escape box that was recorded, and it was analyzed by using ImageJ software (https://imagej.net/downloads; accessed on 10 February 2021; LOCI, University of Wisconsin).

### 4.7. Acoustic Startle Reflex

For testing the acoustic startle reflex, we used the setup developed by us previously [45]. Briefly, a transducer was connected to a perforated Plexiglas box restricting the lateral movements of the animal. Mice were familiar with this Plexiglas box as it was placed in the cage for 1 week and they often used it as shelter. A continuous 60 dB white noise was used whereas the movement of the mouse was checked with a camera. For eliciting acoustic startle reaction and detecting short-term habituation, a 105 dB noise was employed 5 times with 1 min gaps. Amplitudes of the first startle reaction and the average of 5, as well as the short-term habituation, were checked. Short-term habituation is defined as a decrease in the amplitude in the 5 measured responses [58]. Before testing, an extra experimental session was performed to familiarize mice with the experimental environment. All experiments were repeated twice and the averages of the 2 experiments were assessed.

### 4.8. Footprint Test

To assess muscle tone and gait, a footprint test was conducted. The setup included an empty white paper placed on a clean smooth surface, an escape box positioned 50 cm away from the starting point and 2 cardboard walls on both sides of the paper. The result was a one-way path to a shelter with no exits from any other side. Mice were familiar with the shelter box as it was placed in their cage for a week prior to the experiments. Only the middle 30 cm of the path was used for analysis. The trial procedure involved labeling the front paws of the mouse with red food ink and the hind paws with blue food ink (Szilas Maxcolor, Szilasfood, Kistarcsa, Hungary). Mice were allowed to run to the shelter at the upper end of the paper. Prior to the trials, a test session was performed to familiarize the mice with the experimental arrangement. Stride length, sway length and stance length of the front and hind paws were determined in cm [59].

### 4.9. Immunohistochemistry and Post Hoc Analysis

To assess the injection site and the mCherry labeling, as well as the potential changes in neuronal and astrocytic numbers after the CNO treatment in the two groups, mice were transcardially perfused after the last behavioral test. After the fixation, free-floating 70 µm thick coronal mesopontine slices were cut with a vibratome (Campden Instruments, Loughborough, UK). After sectioning, the samples were washed with TBS (3 × 10 min). Blocking and permeabilization were applied using 0.1% Triton X-100 and 10% normal serum (depending on the type of secondary antibody applied) for 1 h at room temperature. Certain slices were labeled with anti-GFAP antibody (rabbit, 1:1000, Synaptic Systems GmbH, Goettingen, Germany) or NeuN-specific (rabbit, 1:1000, EMD Millipore Corporation, Burlington, United States) labeling with blue secondary antibody (Goat anti-Rabbit IgG (H+L) Cross-Adsorbed Secondary Antibody, Alexa Fluor™ 405, 1:1000, Thermo Fisher Scientific, Eugene, OR, USA) to validate the colocalization of mCherry expression and GFAP immunopositivity, whereas others were used for cell counting. For the preliminary experiments on ChAT-tdTomato mice, NeuN-specific labeling was used with green fluorescence (Anti-Rabbit IgG (H+L), highly cross-adsorbed, CF™ 488A antibody produced in donkey, 1:1000, Merck KGaA, Darmstadt, Germany). For the wild-type mice, NeuN with blue fluorescence and choline acetyltransferase-specific (goat, 1:200, EMD Millipore Corporation, Burlington, VT, United States) immunohistochemical labeling was administered using a secondary antibody with green fluorescent dye. The slices were incubated with primary antibodies for 48 h at 4 °C, then the samples were washed with TBS (3 × 10 min). After the washing step, the secondary antibodies were applied for 24 h at 4 °C, then the samples were rinsed with TBS (3 × 10 min). Confocal images were taken with an AiryScan 880 laser scanning confocal microscope (Zeiss, Oberkocken, Germany) equipped with a ×20 objective. GFAP immunohistochemistry and detection of mCherry expression were used to evaluate the injection site. Cell counting was achieved in the coronal plane of the injection in a 0.43 × 0.43 mm square with the center in the PPN.

### 4.10. Statistics

All data represent mean ± SEM. The normal distribution of datasets was evaluated with normality tests and Student’s *t*-test was applied for assessing statistical significance (level of significance: *p* < 0.05).

## 5. Conclusions

In conclusion, chronic astrocytic activation—likely because of eliciting excitotoxicity—affected the cholinergic cell number in the PPN. In addition to this action, further functional alterations in neuronal functions might also have contributed to the changes seen. Based on our previous in vitro studies, it is likely that an mGluR- and NMDAR-dependent special activity pattern of the mesopontine neural networks was elicited, which led to alterations in the behavioral tests. Movement-related changes in the circadian activity, gait and startle reflex mimicked the brainstem-related symptoms of PSP. In addition to previous models of PSP, we demonstrated that selective astrocytic activation is capable of causing similar symptoms as cholinergic cell depletion in or expression of beta-amyloid. These findings underline the role of astrocytes in the pathogenesis of PSP, potentially demonstrating a therapeutic target for slowing the progress of this disease.

In future experiments, the long-term actions of mesopontine astrocytic overactivation on the structures innervated by the region should be observed, as consequential morphological and functional changes in the target structures might be elicited by the neuronal hyperexcitability and loss of the midbrain. Furthermore, the actions of artificial astrocytic activation and the consequential neuronal depletion should be separated by setting experiments employing acute astrocytic activation of the region.

## Figures and Tables

**Table 1:** The number of experimental animals involved in different behavioral tests.

Groups	Acoustic Startle Test	Activity Wheel Test	Barnes Maze Test	Footprint Test	Drug Preference Test
DREADD (hM3D(Gq) and mCherry expression in astrocytes)	8 (C57BL/6)	6 (C57BL/6)	7 (C57BL/6)	9 (C57BL/6)	4 (ChAT-tdTomato)
Operated control (mCherry expression in astrocytes)	7 (C57BL/6)	6 (C57BL/6)	7 (C57BL/6)	6 (C57BL/6)	-

## Data Availability

Data is contained within the article.
